# Impact of Hydroxy‐Methyl‐Butyrate Supplementation on Malnourished Patients Assessed Using AI‐Enhanced Ultrasound Imaging

**DOI:** 10.1002/jcsm.13700

**Published:** 2025-02-24

**Authors:** Daniel de Luis, Angela Cebria, David Primo, Sara Nozal, Olatz Izaola, Eduardo Jorge Godoy, Juan Jose Lopez Gomez

**Affiliations:** ^1^ Centro de Investigación of Endocrinología, Nutrición Facultad de Medicina, Departamento de Endocrinología Nutrición Hospital Clínico Universitario de Valladolid Valladolid Spain; ^2^ DAWAKO Medtech SL Parc Cientìfic de la Universitat de Valencia Paterna Valencia Spain; ^3^ Técnicas Avanzadas de Desarrollo de Software Centrado en la Persona, Departamento de Informática Universitat de Valencia Burjassot Valencia Spain

**Keywords:** artificial intelligence, disease‐related malnutrition (DRM), hydroxy‐methyl‐butyrate (HMB), oral nutritional supplement (ONS), ultrasound‐imaging

## Abstract

**Background:**

This study aimed to evaluate the effects of an oral nutritional supplement (ONS) enriched with hydroxy‐methyl‐butyrate (HMB) in subjects with disease‐related malnutrition (DRM) and to monitor these effects with an ultrasound Imaging System, based on artificial intelligence, in a real‐world study.

**Methods:**

Fifty consecutive adult patients with DRM were enrolled. The malnutrition was diagnosed by Global Leadership Initiative on Malnutrition (GLIM) criteria. For 3 months, the patients received nutritional education, and medical nutrition therapy was started with an adapted oral diet and two servings of an oral nutritional supplementation (ONS) with a hyperproteic hypercaloric formula (HMB—enriched). All patients were studied at baseline, and 3 months after intervention, with a nutritional assessment (anthropometry, bioelectrical impedanciometry [BIA], muscle ultrasonography and biochemical parameters). Ultrasound images were automatically quantified using an AI‐based ultrasound imaging system.

**Results:**

The study included 50 patients (21 men and 29 women) with a mean age of 57.8 ± 18.1 years. Following treatment with the HMB‐enriched ONS, the prevalence of sarcopenia decreased significantly (from 24% to 18%; *p* = 0.01) and severe malnutrition (from 14% to 2%; *p* = 0.01). An improvement in BIA parameters; phase angle (0.14 ± 0.02°; *p* = 0.02); phase angle index (0.07 ± 0.01°/m^2^; *p* = 0.01); fat free mass (1.31 ± 0.12 kg; *p* = 0.006); skeletal muscle mass (0.51 ± 0.11 kg; *p* = 0.01); and skeletal muscle mass index (0.31 ± 0.13 kg/m^2^; *p* = 0.01) were reported. Functional parameters as handgrip strength (1.36 ± 0.21 kg; *p* = 0.009) and seconds of time up and go test (1.16 ± 0.15 s; *p* = 0.02) showed also an improvement. The AI‐based ultrasound system detected a significant increase in the thickness of the vastus intermedius muscle (TVI) (0.09 ± 0.01 cm; *p* = 0.008) and an increase of the rectus femoris (TRF) (1.06 ± 0.4 cm; *p* = 0.07) with the consequence of a significant increase of the (TVITRF) (0.15 ± 0.02 cm; *p* = 0.008). The sarcopenia index is also significant (1.03 ± 0.3 cm; *p* = 0.03). Muscle quality, as assessed by echogenicity analysis, improved significantly: muscle index (Mi) (0.04 ± 0.01; *p* = 0.04) and (fat index) FATi (−0.03 ± 0.01; *p* = 0.03).

**Conclusions:**

The use of an HMB‐enriched ONS in DRM patients, combined with AI‐based ultrasound imaging for follow‐up, significantly improved their nutritional status, muscle mass and muscle quality as assessed by echogenicity.

## 1 Introduction

Therefore, oral nutritional supplements (ONSs) in addition to regular diet are recommended for these patients [1]. Amino acids like leucine found in whey protein and its metabolites such as hydroxy‐methyl‐butyrate (HMB) in oral nutritional supplementation are suggested to be a potential target for improving muscle recovery and function, particularly in older adults [2, 3, 4]. The main mechanisms of the positive effect of HMB on muscles are increasing protein synthesis by stimulating the mammalian target of rapamycin (mTOR) signalling pathway, increasing IGF‐1 serum concentrations, decreasing protein breakdown by downregulating the catabolic signal pathways, enhancing muscle repair by increasing proliferation of satellite cells and decreasing inflammatory mediators and increasing aerobic capacity by improving mitochondrial biogenesis [2, 3].

Whey protein‐based ONS leads to the highest postmeal plasma levels of amino acids and enhances muscle protein synthesis more effectively than other types of proteins. Certain whey protein‐based ONSs contain high levels of leucine, an amino acid that plays a key role in muscle growth.

NOURISH study reported that patients with DRM (cardiac failure, myocardial acute infarction and COPD) who were treated with an HMB‐enriched hypercaloric hyperproteic ONS had a lower mortality rate at 90 days than control [5]. This study raised the possibility of the effect of oral nutrition supplementation over the complications of malnutrition. Real World studies, as a study performed in routine clinical practice, with ONS enriched in leucine or its metabolites are scarce [6, 7].

Disease‐related malnutrition (DRM) is commonly acknowledged as a widespread and significant issue in various health and social care environments [8]. It is typically identified by changes in body structure, such as the breakdown of muscle mass and loss of lean body mass, functional limitations and negative clinical results in individuals dealing with long‐term illnesses or sudden episodes of disease [9, 10].

DRM prevalence in outpatient settings varies from 21% to 69%, depending on the underlying condition and assessment methods used [11, 12, 13]. DRM is linked to a variety of negative health and economic consequences. Patients with DRM usually experience decreased functional abilities, such as reduced muscle strength, limited physical performance and significant limitations in everyday activities [14, 15, 16]. Muscle mass measurement is considered an important factor in diagnosing disease‐related malnutrition, as outlined by the Global Leadership Initiative on Malnutrition (GLIM) [17].

The European Working Group on Sarcopenia in the Elderly [18] has released a consensus stating that low muscle strength is now considered the primary characteristic of the condition, rather than low muscle mass. ESPEN guidelines recommend an energy intake of 30 kcal/kg body weight/day and a higher protein intake for older adults and patients with DRM [19]. For these groups, it is advised to consume 1.2–1.5 g/kg body weight/day of protein (equivalent to 25–30 g protein/meal) with high protein quality to fulfil nutritional needs and enhance muscle synthesis [20].

In this setting, nutritional ultrasound, which assesses muscle mass, is a developing and cost‐effective method that measures this tissue in malnourished individuals. It offers advantages over expensive and less accessible healthcare techniques such as computed tomography, magnetic resonance imaging or dual photon X‐ray absorptiometry [21].

The main problem of muscle ultrasound is the great interobserver variability that exists. Therefore, automatic reading systems based on artificial intelligence and machine learning algorithms can help homogenize the results obtained with muscle ultrasound and can be appropriately related to the readings made by the radiologists on the original image [22].

This study aimed to evaluate the effects of an ONS enriched with HMB in subjects with DRM and to monitor these nutritional effects with an ultrasound imaging system based on artificial intelligence in a real‐world study.

## 2 Materials and Methods

### 2.1 Procedures

Fifty consecutive adults with malnutrition related to disease were deemed eligible if they had received a DRM diagnosis during their visit to our Nutritional Unit and provided signed informed consent. Exclusion criteria included liver dysfunction (aminotransferase levels > 3 times the upper reference limit), chronic renal failure (glomerular filtration rate < 45 mL/min/1.73 m^2^), previous ICU stay during the last hospital admission, cancer patients with an Eastern Cooperative Oncology Group performance status ≥ 3 points, eating disorders, any musculoskeletal disease preventing unassisted walking ability, dementia, cognitive impairment or any neurological/psychiatric condition that could interfere with study procedures; life expectancy of less than 6 months; and refusal to sign the informed consent form. Malnutrition was evaluated using the Global Leadership Initiative on Malnutrition criteria [17]. The protocol (Code PIP22‐2559) was authorized by the Ethics Committee for Clinical Research of the Health Council of HCUVA. All patients participating in the study provided written informed consent. Sociodemographic parameters, anthropometric characteristics, SARCF test, handgrip strength, bioimpedance analysis, the Timed Up and Go test, and ultrasound of rectus femoris (RF) and vastus intermedius (VI) were conducted on all patients, at basal time and after 3 months of dietary intervention with two servings of an enriched HMB ONS; see Table 1.

**TABLE 1:** Composition of oral nutritional supplement enriched with hydroxy‐beta‐methyl butyrate (HMB).

1 unit 220 (ml)	
Total energy (Kcal)	330
Protein (g)	18 (22.14%)
Total lipid (g)	11 (28.81%)
HMB (g)	1.5
Vitamin D3 (μg)	12
Carbohydrate (g)	39 (46.95%)
Dietary fibre (g)	1.7

*Note:* Dietary fibre source: oligofructose.

Abbreviation: HMB, hydroxy‐beta‐methyl butyrate.

### 2.2 Nutritional Intervention

For 3 months, the patients received nutritional education, and medical nutrition therapy was started with an adapted oral diet with specific food enrichment measures for patients with disease related malnutrition and two servings of an oral nutritional supplementation with an hyperproteic hypercaloric formula (1.5 kcal/mL, 9.1 g protein/100 mL, 16.8 g carbohydrates/100 mL, 4.8 g fats/100 mL and 0.68 g HMB/100 mL) (Ensure Plus Advance®) with 220 mL per bottle (Table 1); this represents a total daily amount of HMB of 3 g/day. In our design, there was no control group versus the group that received treatment.

Patients recorded daily supplement intake in a questionnaire and received telephone calls every 4 weeks to reinforce adherence to treatment. The presence of adverse effects with taking ONS enriched in HMB was also recorded.

### 2.3 Determinations

All the parameters were measured at basal condition before intervention and after 3 months of intervention. Height (cm) and waist circumference (cm) were measured with a nonelastic measuring tape (Omrom, LA, CA, USA). Body weight was determined with the subjects unclothed, using a digital scale (Omrom, LA, CA, USA). Using these parameters, body mass index (BMI) was calculated (body weight [kg] divided by the square of height [m]). Fat mass (FM) and skeletal muscle mass (SM) were determined by bioimpedance with a precision of 5 g [23] (EFG BIA 101 Anniversary, Akern, It), using the following equation for its calculation (0.756 height^2^/resistance) + (0.110 × body mass) + (0.107 × reactance) − 5463.

Screening for sarcopenia risk was evaluated using the Strength, Assistance with walking, Rising from a chair, Climb stairs, Falls (SARC‐F) questionnaire, with a score ≥ 4 indicating sarcopenia risk. Handgrip strength was measured with the Jamar dynamometer (J. A. Preston Corporation, New York, NY, USA) on the dominant hand. Three measurements were taken and averaged and compared against EWGSWOP2 cut‐off points to determine probable sarcopenia. Sarcopenia assessment followed EWGSOP2 criteria to identify confirmed cases based on probable sarcopenia criteria plus abnormal (skeletal muscle index) SMI detected through BIA (< 7.0 kg/m^2^ for men, and < 5.5 kg/m^2^ for women) [11]. The functionality was assessed using the time up and go (TUG) test. An armless chair served as the starting point, with coloured tape placed 3 m away. Participants were required to walk to the tape, turn around and return to the chair at their fastest pace. A timer began when they stood up from the chair and stopped when seated again. Practice trials were conducted before the actual test. A TUG‐score of ≥ 20 s was established as indicative of severe sarcopenia [18].

Ultrasound assessments in the dominant leg of the unilateral right rectus femoris muscle (RF) and vastus intermedius muscle (VI) were performed using a portable ultrasound system with a 4‐ to 10‐cm linear probe (UProbe L6C Ultrasound Scanner, Guangzhou Sonostar Technologies Co., Ltd., Guangzhou, Guangdong, P.R. China). The measurements were taken on the anterior thigh region while the patient lays supine with extended and relaxed knees. The acquisition site was located two thirds along the length of the femur, between the anterior superior iliac spine and upper edge of patella.

**FIGURE 1:**
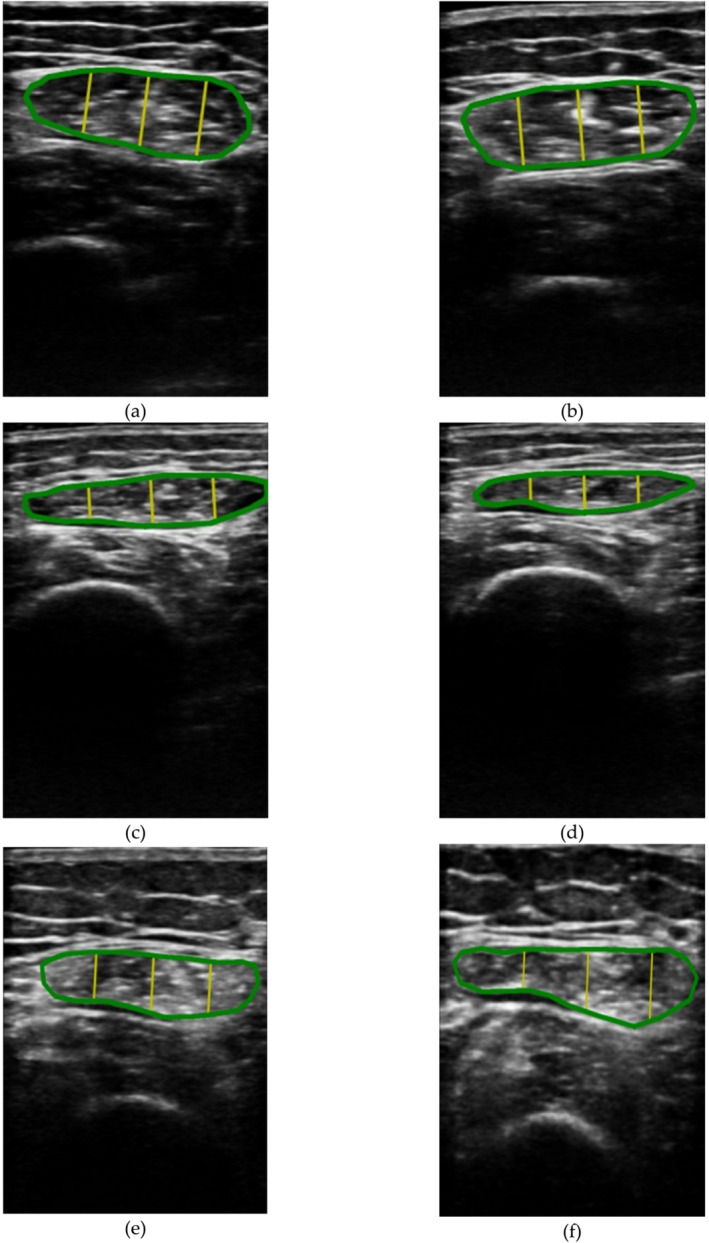
The figure shows an example of three cases of the acquired ultrasound images of the rectus femoris muscle in the transverse plane (automatic segmented ROI of the muscle belly in green colour, and the yellow lines indicate the three measurements of the muscle thickness to compute its mean thickness value). Images (a), (c) and (d) are the acquired images and thickness measurement previous the treatment with the ONS enriched with HMB. The images (b), (d) and (f) are the acquisition and thickness measurements, of the same patients, post treatment with the ONS enriched with HMB. The average of the three yellow marks of (d)–(f) were superior to (a)–(c), respectively.

The acquired ultrasound images were processed and analysed using the automatic system based on artificial intelligence (PIIXMED™) (DAWAKO Medtech S.L., Valencia, Spain). Figure 1a,c,e shows three examples of the ultrasound images acquired at basal condition before intervention, and Figure 1b,d,f shows the respective ultrasound images after 3 months of intervention. The cloud‐based web application software utilizes convolutional neural networks (CNNs) [22], with a U‐net architecture, designed for the automatic segmentation of regions of interest (ROI), in green colour. The yellow lines correspond to the three measurements of muscle thickness, which are averaged to obtain the quantification of the muscle thickness. The U‐Net is a convolutional neural network for biomedical image segmentation, which will receive images as input and return segmentation maps as output. The network architecture was designed to work with fewer training images and produce more accurate segmentations than previous proposals. The network architecture consists of a first part of contraction followed by a second part of expansion. The contraction part consists of the repeated application of two 3 × 3 convolutions, each followed by a rectified linear unit (ReLU) and a max 2 × 2 pooling operation with Stride 2 for resolution reduction. Finally, the software shows titckess of both muscle and the quality indeces based on histrogram fragmentation of echo‐intensity.

With this system, we recorded thickness of the muscle rectus femoris (TRF), the thickness of the vastus intermedius muscle (TVI) and the thickness of both muscles (TVI + TRF).

All these variables were standardized by dividing them by the square of the height in meters, obtaining the TRFi, TVIi and the TRFTVIi, all of them described at Table 4.

**FIGURE 2:**
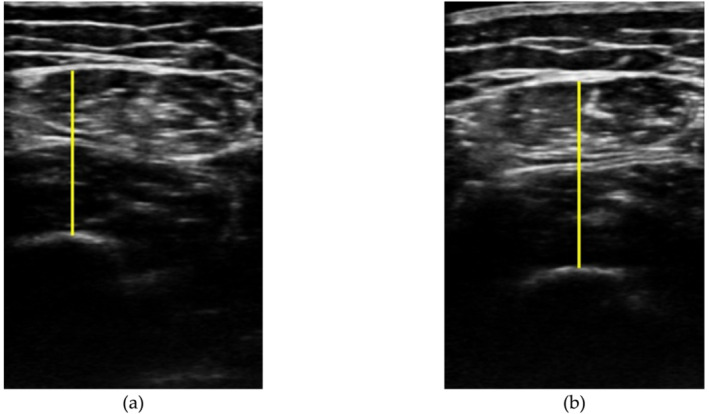
Segmentation and quantification of the thigh thickness by PIIXMED™ (i.e., the thickness from the upper aponeurosis to the surface of the femur bone—yellow line). (a) Example of the SARCOPENIA index (STAR) (i.e., dividing the thigh thickness by the BMI) from a patient previous the treatment with the ONS enriched with HMB @ (STAR = 1.0); (b) the same patient post treatment with the ONS enriched with HMB @ (STAR = 1.1). This index has increased by 0.1 points after nutritional intervention.

The TVI + TRF will serve as the required quantification of the thigh thickness (i.e., the thickness from the upper aponeurosis to the surface of the femur bone) for the SARCOPENIA index (STAR) implemented in the ultrasound automated system based on AI (PIIXMED™). Figure 2 shows the automatic segmentation of the thigh. The calculated SARCOPENIA index is obtained by dividing the thigh thickness by the BMI, following the formula provided by Kara et al. [24], where a value less than 1.4 in men and 1.0 in women confirms sarcopenia.

The following muscle quality indices based on echo‐intensity (i.e., histogram–base) were evaluated: the muscle to no muscle index (Mi), which is the proportion of lean muscle content, and the Fat to muscle index (FATi), which is the proportion of interstitial fat content.

At the initial and after 3 months of nutritional intervention, fasting blood samples were drawn for measurement of C‐reactive protein (< 3 mg/dL), albumin (3.5–4.5 g/dL), prealbumin (18–28 mg/dL) and transferrin (250–350 mg/dL) (Hitachi, ATM, Manheim, Ger).

### 2.4 Statistical Analysis

A power calculation based on weight improvement was performed. Fifty patients were necessary to detect an improvement of 0.5 kg of muscle mass, with an error type *I* < 0.05 and a statistical power of 80%. The sample size calculation was performed with the increase in muscle mass, because the objective of the work was to evaluate the effect of an HMB‐enriched ONS in a patient with DRM, monitored by muscle ultrasound. Statistical tests were two‐tailed and conducted at the 0.05 significance level, and *p* values were rounded to four decimal places. Quantitative variables with normal distribution were analysed with two tailed paired or unpaired Student's *t* test. Nonparametric variables were analysed with Wilcoxon test. The statistical package used was SPSS 23.0, IL, USA.

## 3 Results

Our sample was made up of 50 patients (21 men and 29 women), with a mean age of 57.8 ± 18.1 years. Anthropometric, biochemical and BIA parameters are shown in Table 2. All patients had DRM, with a positive phenotypic and etiologic criterion as indicated by GLIM criteria (10). The distribution of diseases were *n* = 19 of patients presents with benign digestive tract pathology (inflammatory bowel disease and other pathologies that cause malabsorption), *n* = 13 with nonterminal oncological pathology, *n* = 5 with chronic neurological pathology and *n* = 13 with chronic heart‐lung pathology. A total of 7 patients had severe malnutrition by GLIM criteria (14%) at the time of diagnosis. Completion of ONS intake was 93%. No patient reported any adverse effects in relation to taking ONS enriched in HMB.

**TABLE 2:** Anthropometric, bioimpedanciometry, functional variables and biochemical parameters (means ± SD), before and after nutritional treatment with an ONS enriched with HMB.

Parameters	Basal	3 months	*p* value
**Anthropometric parameters**			
BMI (kg/m^2^)	20.83 ± 3.58	21.10 ± 3.56	0.20
Weight (kg)	55.40 ± 11.31	55.80 ± 11.40	0.18
Calf Circumference (cm)	32.5 ± 3.46	32.6 ± 3.41	0.23
**BIA parameters**			
Resistance (ohm)	591.0 ± 87.1	584.6 ± 100.11	0.23
Reactance (ohm)	52.9 ± 11.30	53.54 ± 12.21	0.25
Phase angle (°)	5.14 ± 1.01	5.28 ± 1.07	0.02
Phase angle index (°/m^2^)	1.94 ± 0.41	2.01 ± 0.41	0.01
Fat mass FM (kg)	12.01 ± 6.50	11.40 ± 6.31	0.33
Fat free mass FFM (kg)	42.94 ± 8.81	44.11 ± 8.72	0.01
SM (kg)	16.00 ± 3.61	16.5 ± 4.13	0.01
SMI (kg/m^2^)	5.9 ± 0.90	6.2 ± 1.12	0.01
**Functional parameters**			
Time up and go (sg)	13.57 ± 5.41	12.40 ± 4.30	0.04
Handgrip Strength (kg)	22.20 ± 3.56	23.56 ± 9.51	0.01
**Biochemical parameters**			
Albumin (g/L)	4.33 ± 0.41	4.36 ± 0.52	0.31
Prealbumin (g/L)	22.3 ± 5.91	22.8 ± 3.14	0.33
C Reactive protein (g/dL)	5.0 ± 0.92	3.9 ± 1.19	0.21

Abbreviations: BIA, bio impedanciometry; BMI, body mass index; SM, skeletal muscle mass; SMI, skeletal muscle index.

The SARC‐F questionnaire reported a 64% (*n* = 32) of the patients at risk of sarcopenia (> 4 points). Handgrip strength showed 40% (*n* = 20) of the patients with probable sarcopenia, as indicated by the dinapenia status (< 27.0 kg for men and < 16 kg for women) (Table 3). Appendicular skeletal muscle mass (SMI) (kg) by BIA reported 24% (*n* = 12) with confirmed sarcopenia (< 7.0 kg/m^2^ for men and < 5.5 kg/m^2^ for women). A TUG‐score of ≥ 20 s was identified as a cut‐off point for severe sarcopenia and reported 12% (*n* = 6) of the patients.

**TABLE 3:** Malnutrition and sarcopenia ratios before and after nutritional intervention with an ONS enriched with HMB.

Parameters	Baseline	After 3 months	*p* value
Severe malnutrition	14%	2%	0.01
% Risk sarcopenia (SARCF ≥ 4)	64%	4%	0.01
% Probable sarcopenia	40%	30%	0.01
% Confirmed sarcopenia	24%	18%	0.01
% Severe sarcopenia	12%	6%	0.01

After dietary intervention, only one patient (2%) showed severe malnutrition and 98% moderate malnutrition. The percentages of different status of sarcopenia improved, too. The SARC‐F questionnaire reported a 4% (*n* = 2) of the patients at risk of sarcopenia (> 4 points). Handgrip strength showed 30% *n* = 15 of the patients with probable sarcopenia. Confirmed sarcopenia (< 7.0 kg/m2 for men and < 5.5 kg/m2 for women) was detected in 18% (*n* = 9), and severe sarcopenia was reported 6% (*n* = 3) of the patients. Table 3 shows the improvement in these percentages.

Table 2 shows the enhancement in BIA parameters: phase angle (0.14 ± 0.02°; *p* = 0.02), phase angle index (0.07 ± 0.01°/m^2^; *p* = 0.01), fat free mass (1.31 ± 0.12 kg; *p* = 0.006), skeletal muscle mass (0.51 ± 0.11 kg; *p* = 0.01) and skeletal muscle mass index (0.31 ± 0.13 kg; *p* = 0.01). Functional parameters as handgrip strength (1.36 ± 0.21 kg; *p* = 0.009) and seconds of time up and go test (1.16 ± 0.15 sg; *p* = 0.02) improved, too. Some significant increases in BIA parameters have a clinically insignificant translation, because they represent less than 10% of the total value, for example, phase angle.

Table 2 reports classical anthropometric parameters (body weight and calf circumference); all these parameters did not show changes.

Finally, albumin, prealbumin and C Reactive protein levels remained unchanged (Table 2).

Table 4 shows the ultrasound parameters. The following parameters of ultrasound images showed a significant improvement: thickness of the muscle vastus intermedius (TVI) (0.09 ± 0.01 cm; *p* = 0.008), thickness of the muscle vastus intermedius plus rectus femoris (TVITRF) (0.15 ± 0.02 cm; *p* = 0.008) and thickness of the muscle vastus intermedius index TVIi (0.03 ± 0.01 cm/m2; *p* = 0.03). Muscle quality, as assessed by echogenicity analysis, improved significantly; muscle index (Mi) (0.04 ± 0.01; *p* = 0.04), and (fat index) FATi (−0.03 ± 0.01; *p* = 0.03). Lastly, the SARCOPENIA index (STAR) also increased (0.05 ± 0.02; *p* = 0.03).

**TABLE 4:** Differences in the image parameters (means ± SD) by an ultrasound imaging system based on artificial intelligence before and after nutritional treatment with an ONS enriched with HMB.

Ultrasound parameters	Basal	3 months	*p* value
TRF (cm)	1.01 ± 0.31	1.06 ± 0.43	0.07
TVI (cm)	1.00 ± 0.41	1.09 ± 0.51	0.01
TVITRF (cm)	2.01 ± 0.75	2.16 ± 0.72	0.01
TRFi (cm/m^2^)	0.33 ± 0.12	0.34 ± 0.21	0.21
TVIi (cm/m^2^)	0.37 ± 0.12	0.41 ± 0.11	0.01
TVITRFi (cm/m^2^)	0.37 ± 0.12	0.40 ± 0.43	0.10
SARCOPENIA index (STAR)	0.98 ± 0.30	1.03 ± 0.31	0.03
Mi	0.62 ± 0.15	0.66 ± 0.13	0.04
FATi	0.29 ± 0.13	0.26 ± 0.72	0.03

*Note:* SARCOPENIA index (STAR) [24] (thigh thickness [TVITRF]/BMI).

Abbreviations: FATi, fat to muscle index (FATi); Mi, muscle to no muscle index; TRF, thickness of the muscle rectus femoris; TRFi, thickness of the muscle rectus femoris index; TVI, thickness of the vastus intermedius muscle; TVIi, thickness of the vastus intermedius muscle index; TVITRF, thickness of both muscles; TVITRFi, thickness of both muscles index.

**FIGURE 3:**
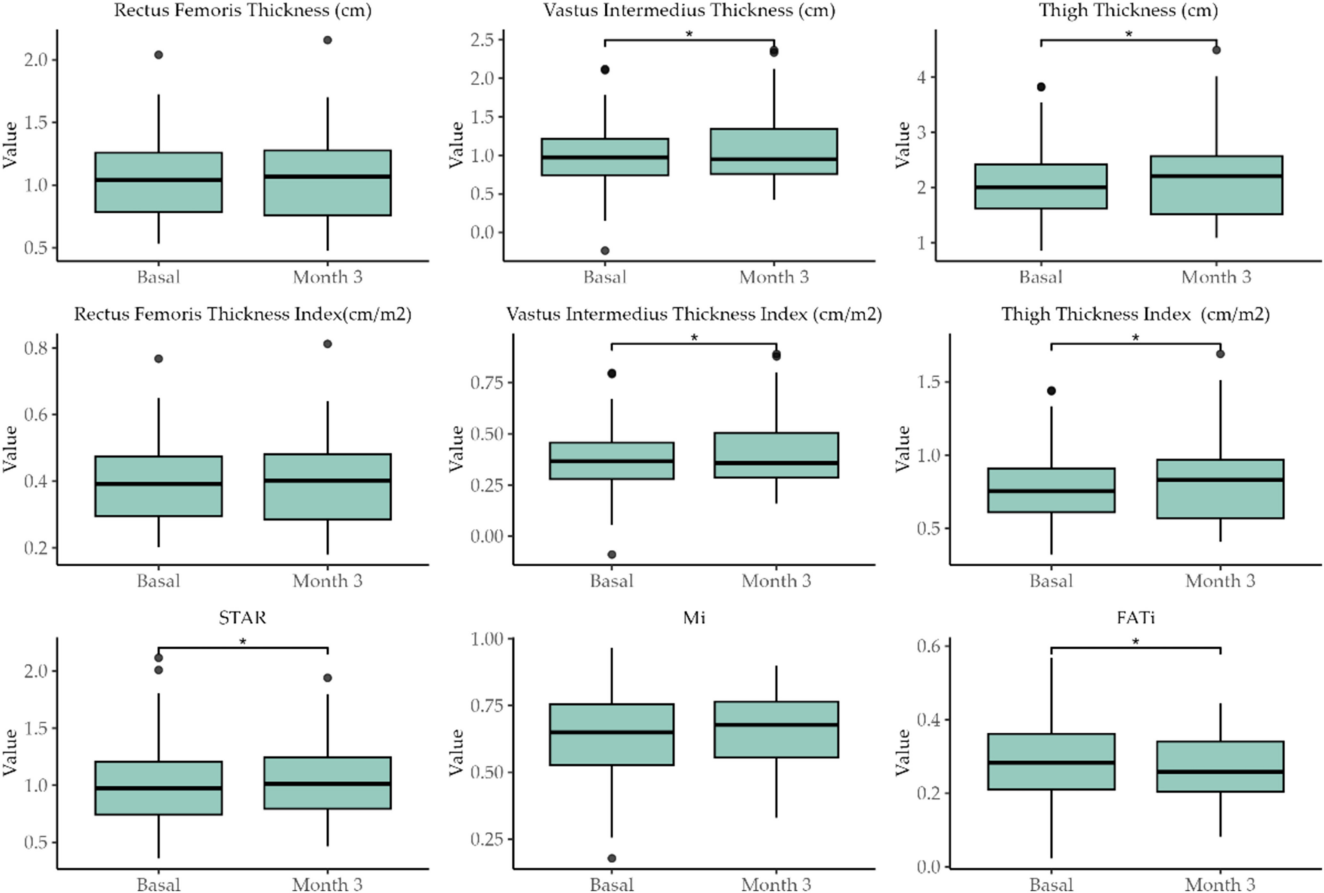
Boxplots of the image variables shown in Table 4. The inset asterisk above the compered boxplots indicates a significant difference between previous (basal) and posttreatment (3 months of treatment) with the ONS enriched with HMB.

Figure 3 shows the boxplots of the image variables shown in Table 4. The inset asterisk above the compared boxplots indicates a significant difference between previous (basal) and post treatment (3 months after treatment) with the ONS enriched with HMB.

## 4 Discussion

Our study demonstrated that an ONS enriched with HMB increase muscle mass and strength in outpatients with DRM, with a decrease in the percentage of patients with sarcopenia after 3 months of treatment and the patients presented better muscle quality indexes based on histogram echo‐intensity.

Skeletal muscle decrease and weakness commonly occur with malnutrition and age‐related sarcopenia [1, 3, 4]. These factors are strongly related to impaired physical function [8] which produce morbidity and mortality [25]. Thus, nutritional interventions that can reduce or even prevent muscle mass loss and enhance physical function is a crucial clinical priority. The measurement or estimation of skeletal muscle can vary based on the technique employed, categorizing it as either fat‐free mass (FFM) or lean mass, although the accuracy can differ. For instance, bioelectrical impedance analysis (BIA) provides an estimate of FFM, which includes the sum of lean body mass and bone mineral compartments. On the other hand, dual‐energy X‐ray absorptiometry (DXA) measures lean mass, encompassing body water, total body protein, carbohydrates, nonfat lipids and soft tissue mineral [26]. Ultrasound imaging has supplanted or supplemented numerous radiographic and nuclear medicine procedures, paving the way for new diagnostic investigations. This is particularly significant in assessing patients with DRM by examining both the quality and quantity of muscle [27]. Ultrasound technology is fast, it does not use ionizing radiation, and it is a portable technique; the only defect may be interobserver variability; however, our group has demonstrated the high reliability of automatic reading systems based on artificial intelligence [22].

Ultrasound technology is a fast and portable technique; its main advantage is that it does not use ionizing radiation, which makes this technique safe for patients. Despite these, interobserver variability in identifying and analysing ultrasound images is the main drawback of this technique; therefore, automatic reading systems based on artificial intelligence, such as PIIXMED™, have demonstrated high reliability between evaluators [22].

To improve muscle mass in patients with DRM, it is necessary to implement a nutritional approach, increasing caloric and protein intake. However, muscle mass is preserved through a balance between muscle protein synthesis and muscle protein breakdown. Although both resistance exercise and amino acid supplementation can improve protein balance, resistance exercise is particularly difficult during illness as our enrolled patients with different pathologies [28]. Consequently, there is a need to explore new interventions, such as amino acids and their metabolites, which do not solely depend on factors related to appetite. In this context, HMB has been evaluated in different settings as HIV patients [29], patients with cancer [30] and critical illness patients [31]. Recently, a systematic review has demonstrated the effects of HMB on skeletal muscle mass and muscle function, as it is showing in our real‐world study. HMB is a metabolite of the amino acid leucine, and this molecule has different mechanism of actions in order to improve muscle, for an example, attenuation of the proteosome pathways and the autophagy–lysosome system that led to muscle protein [32], stimulation of the mammalian target of rapamycin (mTOR) and the growth hormone/IGF‐1 axis, which leads to increase protein synthesis [33]. In our design, the following ultrasound imaging parameters showed significant improvement: thickness of the muscle vastus intermedius (TVI) and thickness of the muscle vastus medialis plus rectus femoris (TVITRF). An improvement in parameters of muscle echo intensity quality muscle index (Mi), muscle to no muscle index (NMi) and fat index (FATi) were detected, too. All these results of quantitative and qualitative improvement obtained by ultrasound, and also by BIA, support the hypothesis of muscular anabolism produced by ONS enriched in HMB, as indicated by previous studies [29, 30, 31, 32]. Interestingly, this increase in muscle mass reported in our current study and previous above‐mentioned designs was reported without changes in body weight; this fact has not a clear mechanistic effect of HMB supplementation itself and should be interpreted as a potential methodologic problem related with compartmental body composition in all the studies. It is important to highlight those interventions that target muscle protein turnover, in terms of enhancing muscle protein synthesis and reducing muscle protein breakdown, such as ONS enriched with HMB; they are of high clinical interest for daily practice with patients with DRM [34]. In the literature, the studies [32] varied in the intervention design: HMB in combination with arginine and glutamine, HMB alone and HMB in ONS as our current design. However, all these studies have one point in common, and that is the daily dose of HMB, in all 3 g/day, similar to the proposal in our work. Therefore, for future studies, we recommend this minimum effective dose of 3 g/day. This active metabolite of leucine (HMB) has a low conversion rate from the amino acid; it is low that only approximately 5% and administration in ONS should be considered when used in patients with DRM and decreased muscle mass.

Additionally, this is the second study in the literature that demonstrates a decrease in fat in the muscle after the use of HMB. In a previous study, Peng et al. [35] demonstrated a decrease in intramuscle fat in prefrail elderly patients after using the same ONS as our current study. However, the investigators [35] used magnetic resonance imaging (MRI) to assess fat in the muscle, a more expensive and time‐consuming technique than the artificial intelligence system for reading the ultrasound image used in our study. This fat is the ectopic deposit of adipose tissue within the muscle, and higher amounts of this ectopic tissue were associated with insulin resistance, decrease muscle strength and mobility disfunction [36]. Older adults [36] with higher ratios of fat in muscles may have muscle weakness and decrease mobility. Finally, fat is correlated with insulin levels and fasting glucose [37], which remarked the important role of intramuscular fat in metabolism, and the need to begin to determine it in the nutritional assessment of our patients. There is no clear hypothesis for this improvement of fat, but we can imply at least two theories, a decrease in inflammation generated by HMB or even an improvement in the oxidation of fatty acids in the muscle secondary to the activation of enzymes generated by HMB. In our current study, a decrease in intramuscular fat FATi and an increase in muscle mass were detected.

Of interest, the effect of HMB supplementation on strength showed a clear relationship between nutritional status, muscle quality, muscle mass and physical function. In our study, we have seen an improvement in the functionality of the upper extremities with an improvement in the strength determined with dynamometry and, of the lower extremities, demonstrated with a decrease in seconds spent in the time up and go test (TUG). For example, a 1‐kg increase in hand‐grip strength was associated with decreases odds of hospitalization and for falls [38]. Without a doubt, these findings of functional improvement are very interesting, because other studies such as the NOURISH trial have demonstrated an improvement in the survival of patients who consume ONS enriched with HMB for 90 days [18]. Our results and those found in the NOURISH study [18], although they are only after a 3‐month intervention, certainly allow us to think that longer interventions can generate more substantial improvements in nutritional status and muscle mass and therefore in a decrease in complications and functional improvement.

Our study has limitations. First, the duration of the intervention is short 3 months; however, the improvement is significant in muscle mass and functionality. Longer studies are certainly necessary. Moreover, this fact affects our ability to discern the impact of intervention on readmission and survival. Second, the use of ultrasound is a novel technique that has shown evidence in the diagnosis of sarcopenia [39] and presents great interobserver variability, but the use of a reading system based on artificial intelligence gives us more accuracy [22]. Third, the absence of a group without treatment, as a control group, may be a problem; however, nutritional supplementation in patients with DRM is mandatory, and it would not be ethically acceptable for them not to receive supplementation. Finally, the study was carried out on Caucasian subjects and with DRM; therefore, it cannot be generalized to the general population. The strength of our study is the evaluation of a heterogeneous group of patients with DRM, which is close to real clinical practice, therefore making the results very generalizable. Another strength is that our study demonstrates the improvement of muscle mass with two techniques such as BIA and ultrasound; this creates more consistency in the data.

In conclusion, the use of an ONS enriched in HMB in patients with DRM improves the nutritional situation, muscle mass and ultrasound quality of the muscle, assessed with an imaging system based on artificial intelligence and BIA. The study showed improvements in functionality over a 3‐month period, though longer term studies are needed to confirm these findings. More studies are necessary to demonstrate the effectiveness of ONS enriched in HMB and to improve the nutritional status of more patients, such as on postoperative immobilization [40], pulmonary disease [41] and prefrail older adults [35].

## Ethics Statement

The study was conducted have therefore been performed in accordance with the ethical standards laid down in the 1964 Declaration of Helsinki and its later amendments, and the study protocol received approval from the Ethics Committee for Clinical Research of the Health Council of HCUVA (protocol code PIP23341, approval date 9 November 2023), as well as from the individual Institutional Review Boards of the participating hospitals.

## Consent

Informed consent was obtained from all subjects involved in the study.

## Conflicts of Interest

The authors declare no conflicts of interest.

## Data Availability

Data are unavailable due to privacy or ethical restrictions.
